# Supplementary material for: Self-Assembled Filament Layers in Drying Sessile Droplets: From Morphology to Electrical Conductivity
Source: Langmuir. 2026 Mar 17;42(12):8592–605. doi: 10.1021/acs.langmuir.5c06611 (PMC13045022; doi:10.1021/acs.langmuir.5c06611)
Supplement: Supplementary file 1 [file la5c06611_si_001.pdf]

Supplementary Material for "Self-assembled filament layers in drying sessile droplets: from morphology to electrical conductivity"

Johannes Schöttner,[1,*] Qingguang Xie,[1,†] Gaurav Nath,[1] and Jens Harting[1,2,‡]

[1] *Helmholtz Institute Erlangen-Nürnberg for Renewable Energy (IET-2), Forschungszentrum Jülich, Cauerstraße 1, 91058 Erlangen, Germany*

[2] *Department of Chemical and Biological Engineering and Department of Physics, Friedrich-Alexander-Universität Erlangen-Nürnberg, Cauerstraße 1, 91058 Erlangen, Germany*

- Number of pages: 2
- Number of figures: 2

Contents

Figures S1-S2

Fig. S1: Nematic order $S_t(\tilde{r})$ and radial distribution $g(\tilde{r})$ versus normalized radial distance $\tilde{r}$ for fully flexible filaments in the reaction-limited $K = 10$ (left) and the diffusion-limited evaporation regime $K \approx 0$ (right). For filament length of $L = 9$ and $L = 11$ and filament concentration of $c = 0.0107$.

Fig. S2: Nematic order $S_t(\tilde{r})$ and radial distribution $g(\tilde{r})$ versus normalized radial distance $\tilde{r}$ for fully flexible filaments in the reaction-limited $K = 10$ (left) and the diffusion-limited evaporation regime $K \approx 0$ (right). For filament concentration of $c = 0.0107$ and $c = 0.0119$ and filament length of $L = 9$.

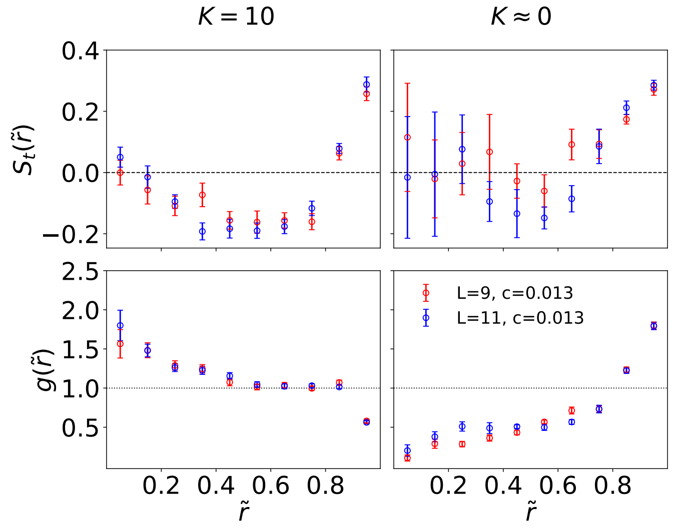

FIG. S1: Nematic order $S_t(\tilde{r})$ and radial distribution $g(\tilde{r})$ versus normalized radial distance $\tilde{r}$ for fully flexible filaments in the reaction-limited $K = 10$ (left) and the diffusion-limited $K \approx 0$ (right) evaporation regime. For filament length of $L = 9$ and $L = 11$ and filament concentration of $c = 0.0107$.

* j.schoettner@fz-juelich.de
† q.xie@fz-juelich.de
‡ j.harting@fz-juelich.de

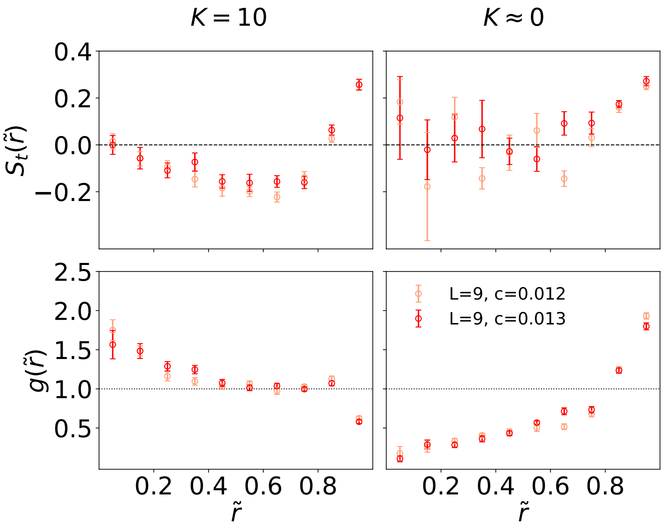

FIG. S2: Nematic order $S_t(\tilde{r})$ and radial distribution $g(\tilde{r})$ versus normalized radial distance $\tilde{r}$ for fully flexible filaments in the reaction-limited $K = 10$ (left) and the diffusion-limited $K \approx 0$ (right) evaporation regime. For filament concentration of $c = 0.0107$ and $c = 0.0119$ and filament length of $L = 9$.
